# Data on the genome analysis of the probiotic strain *Bacillus subtilis* GM5

**DOI:** 10.1016/j.dib.2018.12.081

**Published:** 2018-12-28

**Authors:** G.F. Hadieva, M.T. Lutfullin, D.S. Pudova, Y.A. Akosah, N.E. Gogoleva, E.I. Shagimardanova, A.M. Mardanova, M.R. Sharipova

**Affiliations:** a Department of Microbiology, Institute of Fundamental Medicine and Biology, Kazan (Volga Region) Federal University, Kremlyovskaya str. 18, Kazan 420008 Russia; b Laboratory of Extreme Biology, Institute of Fundamental Medicine and Biology, Kazan (Volga Region) Federal University, Kazan 420021, Russia

**Keywords:** *Bacillus subtilis*, Analysis and assembly of the genome, Antimicrobial lipopeptides

## Abstract

In the present study, we report data on the draft genome sequence of a lipopeptide producing rhizospheric *Bacillus subtilis* GM5 isolate. The genome consists of 4,271,280 bp with a GC-pair content of 43.3%. A total of 4518 genes including 75 tRNA genes, 3 operons coding for rRNA genes and 56 pseudogenes were annotated. Gene clusters responsible for the biosynthesis of secondary metabolites were validated. Six of the thirty-three clusters identified in the genome code for antimicrobial non-ribosomal peptides synthesis. The Whole Genome Shotgun project of *B. subtilis* GM5 has been deposited in the NCBI database under the accession number NZ_NKJH00000000 (https://www.ncbi.nlm.nih.gov/nuccore/NZ_NKJH00000000.1).

**Specifications table**

**Table:** 

Subject area	Biology
More specific subject area	Bioinformatics (Genomics)
Type of data	Table, figures
How data was acquired	Genome sequencing: Illumina Miseq (USA)
	Bioinformatics approaches: NCBI Prokaryotic Genomes Automatic Annotation Pipeline (PGAAP), the RAST web server (http://rast.nmpdr.org/), the antiSMASH server (http://antismash.secondarymetabolites.org)
Data format	Analyzed
Experimental factors	Genomic DNA from pure culture
Experimental features	Isolation of bacteria, genome sequencing, draft genome assembly and annotation, active metabolites prediction
Data source location	*B. subtilis* GM5 was isolated from the potato rhizosphere (Kazan, Russia)
Data accessibility	The whole genome sequence of *B. subtilis* GM5 has been deposited in GenBank under the accession number NZ_NKJH00000000
	(https://www.ncbi.nlm.nih.gov/nuccore/NZ_NKJH00000000.1)
Related research article	« *Bacillus subtilis* strains with antifungal activity against the phytopathogenic fungi» by Mardanova et al. [1]
	«New *Bacillus subtilis* Strains as Promising Probiotics» by Hadieva et al. [2]

**Value of the data**

• The data on the *B. subtilis* GM5 genome is resourceful and can be utilized in understanding their potential biotechnological applications. *B. subtilis* GM5 was concluded to be a promising strain for use as probiotics.
• 33 potential clusters of secondary metabolite synthesis have been identified in the genome of *B. subtilis* GM5 strain, including six gene clusters participating in the biosynthesis of non-ribosomal peptide synthetase (NRPS).
• The data demonstrated here can be used by other researchers working or studying in the field of genome analysis.
• The data presented expands the molecular information on the diversity of *B. subtilis*.

## 1 Data

**Fig. 1:**
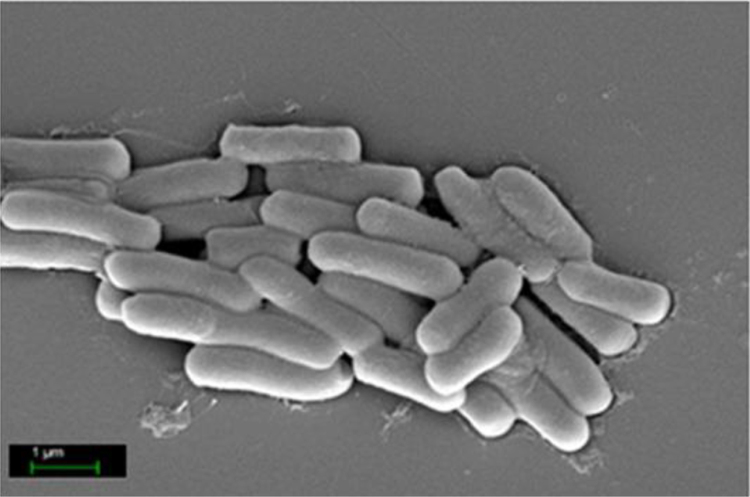
Scanning Electron Microscope of *B. subtilis* GM5.

The *Bacillus subtilis* GM5 isolated from the rhizosphere of potatoes possesses remarkable antimicrobial properties [1] and a probiotic potential [2]. Previous studies have shown that GM5 shows antagonism to pathogenic and opportunistic enterobacteria via the production of cyclic lipo- and dipeptides [1], [2]. The strain was resistant to 1–10% chicken bile and to a wide range of the ambient pH. *B. subtilis* GM5 possessed proteolytic and phytate-hydrolyzing activity and proved to be safe for model animals. Scanning electron microscopy (SEM) of isolated culture showed the presence of rod-shaped cells that are approximately 0.63–0.71 µm in width and 1.80–2.50 µm in length (Fig. 1).

**Fig. 2:**
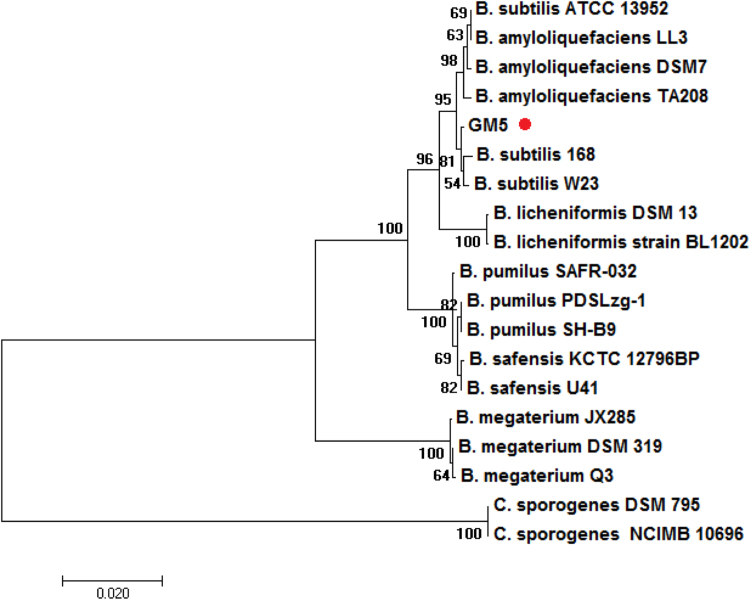
Phylogenetic tree showing the position of *B. subtilis* GM5 relative to other species *Bacillus* strains (GenBank accession numbers for all represented 16 S rRNA sequences are available in Additional file 1). The phylogenetic tree is based on 16 S rRNA gene alignments and was obtained by MEGA 7.0.14 software.

Based on the homology of the 16 S rRNA gene, strain GM5 shares similarity with *B. subtilis* 168 (98% for a 1010 bp sequence). Using a 16 S rRNA based tree, the phylogenetic affiliation of *B. subtilis* GM5 to closely related species within the genus is exhibited (Fig. 2).

The genome of *B. subtilis* GM5 includes 21 contigs, united in 19 scaffolds (N_50_ – 551,988 and L_50_ - 2 bp.). The genome has a size of 4,271,280 bp with a GC-pair content of 43.3%. 4,518 genes were annotated (Table 1), including 4,364 protein coding genes, 75 tRNA genes, 3 operons coding for rRNA genes and 56 pseudogenes. The *B. subtilis* GM5 strain was compared with probiotic strains *Bacillus coagulans* S-lac, *B. subtilis* TO-A JPC [3] and *Bacillus toyonensis* BCT-7112 [4] (Table 1). *B. subtilis* GM5 and *B. subtilis* TO-A JPC share similarity in their primary genomic properties: size, CDS, GC content. (Table 1).

**Table 1:** Comparison of the genomic feature of *B. subtilis* GM5 strain with various *Bacillus* strains. The information regarding the reference genomes was received from PGAAP and the antiSMASH server [12].

Genome feature	*Bacillus subtilis* GM5	*Bacillus subtilis* TO-A JPC [3]	*Bacillus coagulans* S-lac [3]	*Bacillus toyonensis* BCT-7112 [4]
NCBI Accession number	NZ_NKJH00000000.1	CP011882	CP011939	NC_022781.1
Genome size (bp)	4.271.280	4.090.708	3.694.837	5.025.419
GC content (%)	43.3	43.8	46.2	35.5
CDS	4.364	4.231	4.088	4.999
Isolation source	rhizosphere	Vibact®	Sporlac®	soil
Importance of the strain	Probiotic in animal nutrition, biocontrol of plant pathogens	Probiotic for humans and animals	Probiotic for humans and animals	Probiotic in animal nutrition
Identified secondary metabolite clusters	15 (33)	11	1	7

**Fig. 3:**
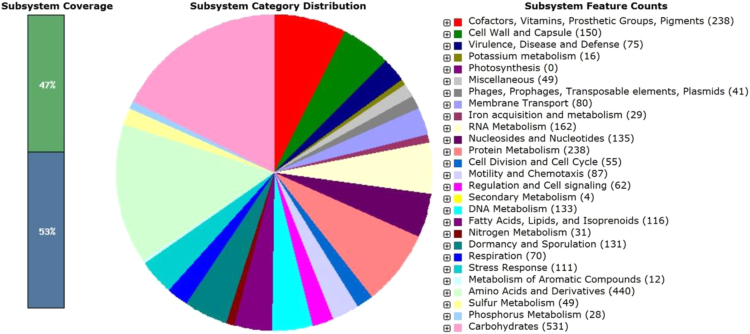
An overview of the subsystem categories assigned to the genome of *Bacillus* subtilis GM5. The whole genome sequence of the strain GM5 was annotated using the Rapid Annotation System Technology (RAST) server. The pie chart demonstrates the count of each subsystem feature and the subsystem coverage.

The RAST server predicted 4,479 coding sequences, of which 2101 coding sequences (47%) were annotated as seed subsystem features and 2378 coding sequences (53%) annotated as outside of the seed subsystem (Fig. 3).

Analysis using the antiSMASH program showed that the genome of GM5 has 33 potential gene clusters, of which 15 (Table 1) are responsible for the synthesis of secondary metabolites, including antimicrobial peptides, terpenes, fatty acids and others. A comparative analysis of the 15 clusters showed 6 gene clusters belonging to the following NRPS family: BGC0001095_c1 Fengycin_biosynthetic_gene_cluster (Compound: Fengycin - possesses antifungal properties [5]), BGC0000407_c1 Plipastatin_ biosynthetic_gene_cluster (compound: plipastatin – a known type of surface-active compound [6]), BGC0001089_c1 Bacillaene_biosynthetic_gene_cluster (Compound: bacillaene - known to exhibit antibacterial activity [7]); BGC0001184_c1 Bacilysin_biosynthetic _gene_cluster (compound: bacilysin - possesses antibacterial activity [8]); BGC0000309_c1 Bacillibactin_biosynthetic_gene_cluster (Compound: bacillibactin – an iron chelator [9]); BGC0000433_c1 Surfactin_biosynthetic_gene_ cluster (compound: surfactin - a known type of surface-active compound [5], [6]) (Table 2). In addition, 18 of the identified clusters were only predicted to be putative.

**Table 2:** NRPS clusters identified in the genome of *B. subtilis* GM5. MIBiG-ID represents the identification number (ID) of cluster in MIBiG (Minimum Information about a Biosynthetic Gene cluster) database. The similarity between the predicted clusters and the clusters in MIBiG is expressed in parentheses.

**Cluster**	**Type**	**Most similar known cluster**	**MIBiG BGC-ID**
1	Nrps	Bacillibactin_biosynthetic_gene_cluster (100% of genes show similarity)	BGC0000309_c1
2	Nrps	Bacillaene_biosynthetic_gene_cluster (100% of genes show similarity)	BGC0001089_c1
3	Nrps	Fengycin_biosynthetic_gene_cluster (100% of genes show similarity)	BGC0001095_c1
4	Nrps	Surfactin_biosynthetic_gene_cluster (82% of genes show similarity)	BGC0000433_c1
5	Nrps	Plipastatin_biosynthetic_gene_cluster (38% of genes show similarity)	BGC0000407_c1
6	Nrps	Bacilysin_biosynthetic_gene_cluster (100% of genes show similarity)	BGC0001184_c1

Thus, the listed gene clusters found in the genome of *B. subtilis* GM5 are responsible for the synthesis of the non-ribosomal cyclic lipopeptides: fengycin, plipastin, surfactin, bacillaene, bacilysin and bacillibactin dipeptide. Thus, an important feature of the *B. subtilis* GM5 genome lies in the fact that much of its genetic material is devoted to the biosynthesis of secondary metabolites. By utilizing these metabolites, *B. subtilis* GM5 is able to suppress pathogenic and conditionally pathogenic microflora, capable of causing intestinal dysbiosis in chickens. The *B. subtilis* GM5 strain has great prospects as a potential probiotic.

## 2 Experimental design, materials and methods

### 2.1 Morphological analysis

The bacterial morphology was investigated using electron scanning microscopy according to the method described in Ref. [10].

### 2.2 Phylogeny analysis

The initial phylogeny of the isolate GM5 was studied using 16 S rRNA analysis. Phylogenetic analysis of the strain GM5 was performed using MEGA 7.0.14 software. Phylogenetic tree was generated using the Maximum likelihood (ML) algorithm with 1000 bootstrap iterations. The strain was deposited in the museum of the laboratory "Biosynthesis and Bioengineering of Enzymes" (Kazan Federal University, Russia).

### 2.3 Genomic DNA preparation

*B. subtilis* strain GM5 was inoculated in 20 ml of LB medium and grown overnight at 37 °C with rocking rate of 200 rpm. 10 mL were centrifuged at 5000×*g* for 10 min at 4 °C and genomic DNA was extracted using Kit NucleoSpin® Microbial DNA. The quality of the final DNA sample were evaluated by gel electrophoresis (1.5% agarose gel) and DNA concentration was estimated by using a NanoDrop 2000с Spectrophotometer (Thermo Scientific). In total, 500 ng/µL of genomic DNA was received and sent for the sequencing.

### 2.4 Genome sequencing and assembly

Whole-genome sequencing of strain GM5 and analysis of genes responsible for the synthesis of antimicrobial peptides were conducted. DNA sequencing was performed using Illumina MiSeq technology by the paired-end method. The quality of the sequencing was checked using the FastQC_v0.11.3. software. De novo assembly and analysis of contigs were carried out using assembler SPAdes_v3.8.1. The statistics of the assembly was calculated with the QUAST_v2.3 program.

### 2.5 Genome annotation

The genome was annotated using the PGAAP NCBI program and the RAST web server [11].

The antiSMASH program (antibiotics & Secondary Metabolite Analysis Shell) [12] was used to analyze the clusters of the antimicrobial metabolites of the GM5 strain, *Bacillus subtilis* TO-A JPC, *Bacillus coagulans* S-lac, *Bacillus toyonensis* BCT-7112. The complete strain genome (FASTA file) was uploaded to the public web version of antiSMASH. The result of the analysis is presented on an interactive HTML page with SVG graphics, and the various parts of the analysis have been displayed in different panels for each gene cluster.
